# Oral contraceptives and menstrual cycle influence autonomic reflex function

**DOI:** 10.14814/phy2.14550

**Published:** 2020-09-05

**Authors:** Elnaz Assadpour, Ilana Ivry, Sara Wasef, Baithat Adeyinka, Kevin R. Murray, Heather Edgell

**Affiliations:** ^1^ School of Kinesiology and Health Science York University Toronto ON Canada

**Keywords:** chemoreflex, hemodynamics, mechanoreflex, metaboreflex, ventilation

## Abstract

Progesterone and its analogues are known to influence ventilation. Therefore, the purpose of this study was to investigate the role of endogenous and pharmaceutical female sex hormones in ventilatory control during the activation of the metaboreflex, mechanoreflex, and CO_2_ chemoreflex. Women aged 18–30 taking (*n* = 14) or not taking (*n* = 12) oral contraceptives (OC and NOC, respectively) were tested in the low hormone (LH) and high hormone (HH) conditions corresponding to the early follicular and mid‐luteal phases (NOC) or placebo and high‐dose pills (OC). Women underwent three randomized trials: (a) 3 min of passive leg movement (PLM), (b) 2 min of 40% maximal voluntary handgrip exercise followed by 2 min of post‐exercise circulatory occlusion (PECO), and (c) 5 min of breathing 5% CO_2_. We primarily measured hemodynamics and ventilation. During PLM, the OC group had a smaller pressor response (*p* = .012). During PECO, the OC group similarly exhibited a smaller pressor response (*p* = .043) and also exhibited a greater ventilatory response (*p* = .024). Lastly, in response to breathing 5% CO_2_, women in the HH phase had a greater ventilatory response (*p* = .022). We found that OC use attenuates the pressor response to both the metaboreflex and mechanoreflex while increasing the ventilatory response to metaboreflex activation. We also found evidence of an enhanced CO_2_ chemoreflex in the HH phase. We hypothesize that OC effects are from the chronic upregulation of pulmonary and vascular β‐adrenergic receptors. We further suggest that the increased cyclic progesterone in the HH phase enhances the chemoreflex.

## Graphical abstract

We have shown that natural and pharmaceutical female sex hormones can influence the metaboreflex, mechanoreflex, and CO_2_ chemoreflex. Oral contraceptives users have attenuated pressor responses to both metaboreflex and mechanoreflex activation concurrent with augmented ventilatory responses to metaboreflex activation. Women in the high hormone phase of the menstrual cycle have a greater ventilatory response to CO_2_ compared to the low hormone phase.

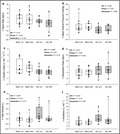

## 1 INTRODUCTION

Exercise activates multiple autonomic reflexes including the mechanoreflex, metaboreflex, and the carbon dioxide (CO_2_) chemoreflex. The mechanoreflex and metaboreflex together form the exercise pressor response, which contributes to the cardiovascular and respiratory response to exercise (Hultman & Sjöholm, 1982; Kaufman & Hayes, 2002). The metaboreflex responds primarily to chemical stimuli, notably metabolite build‐up postexercise (e.g., lactic acid) via unmyelinated group IV afferent fibers (Rotto & Kaufman, 1988; Sinoway, Hill, Pickar, & Kaufman, 1993; Thimm & Baum, 1987), while the mechanoreflex responds primarily to mechanical muscle activity via thinly myelinated group III afferent fibers (Hayes & Kaufman, 2001; Mark, Victor, Nerhed, & Wallin, 1985). Overall, the exercise pressor response results in higher sympathetic output, heart rate (HR), cardiac output (Q), and mean arterial pressure (MAP) (Edgell & Stickland, 2014; Jarvis et al., 2011; Katayama et al., 2018; Llwyd, Panerai, & Robinson, 2017; Vianna, Oliveira, Ramos, Ricardo, & Araujo, 2010). The CO_2_ chemoreflex is also activated during prolonged high intensity exercise above the anaerobic threshold, stimulating respiratory compensation to metabolic acidosis from increased lactate and CO_2_ production (Eldridge, Kiley, & Millhorn, 1985; Rausch, Whipp, Wasserman, & Huszczuk, 1991; Teppema, Barts, & Evers, 1984). This response is governed by the activation of the peripheral and central chemoreceptors, which respond to changes in either arterial CO_2_ or oxygen (O_2_), respectively (Kawai, Ballantyne, Mückenhoff, & Scheid, 1996; Lahiri & DeLaney, 1975). While recent studies have begun to investigate the role of the menstrual cycle and oral contraceptive use in exercise capacity (Keller, Harrison, & Lalande, 2020; Mattu, Iannetta, MacInnis, Doyle‐Baker, & Murias, 2020), their role in isolated autonomic reflex function is still unclear.

There is some evidence that resting sympathetic activity is altered between the early follicular (EF; low levels of hormones) and mid‐luteal (ML; high levels of hormones) phases of the menstrual cycle (Minson, Halliwill, Young, & Joyner, 2000a; Usselman et al., 2015), as well as conflicting reports that OC use may (Usselman et al., 2013) or may not (Carter, Fu, Minson, & Joyner, 2013; Middlekauff, Park, & Gornbein, 2012; Minson, Halliwill, Young, & Joyner, 2000b) affect resting sympathetic activity. Compared to men, women have an attenuated metaboreflex (Ettinger et al., 1996; Jarvis et al., 2011; Joshi & Edgell, 2019), which is unaffected by menstrual cycle phase (Jarvis et al., 2011). Interestingly, sex‐based differences in metaboreflex function are ameliorated in oral contraceptive (OC) users (Minahan et al., 2018) perhaps suggesting a role for female sex hormones. Similarly, the mechanoreflex has been shown to be attenuated in women, compared to men, during passive knee extension (Fouladi, Joshi, & Edgell, 2019; Ives, McDaniel, Witman, & Richardson, 2013), and in premenopausal compared to postmenopausal women during passive ankle dorsiflexion (Park & Kim, 2011); however, no previous work has examined the impact of OC use on mechanoreflex function. Importantly, no previous work has investigated the role of the menstrual cycle or OC use on the ventilatory responses to either metaboreflex or mechanoreflex activation despite the known ventilatory effects of progesterone (Bayliss, Millhorn, Gallman, & Cidlowski, 1987; Bayliss, Seroogy, & Millhorn, 1991). Sex hormones also appear to affect the chemoreflex as Usselman et al. have shown greater sympathoexcitation, yet attenuated neurovascular transduction (Usselman et al., 2015), in the EF compared to the ML phase of the menstrual cycle. Furthermore, chemoreflex regulation appears to be similar in OC users as CO_2_ rebreathing elicits greater sympathoexcitation in the low hormone (LH) compared to the high hormone (HH) phase (Usselman et al., 2013).

The purpose of this study was to assess the affect of menstrual cycle phase and OC use on hemodynamics and ventilation during activation of some of the autonomic reflexes which are in use during exercise. We hypothesized that compared to women not taking OC, the OC group would have: (a) a reduced exercise pressor response and (b) an enhanced ventilatory response to autonomic reflex activation. Furthermore, we also hypothesized that all of the responses would be more pronounced in the HH phase of the menstrual cycle (either ML phase or the high‐dose pill in regularly cycling women and OC users, respectively).

## 2 METHODS

### 2.1 Ethical approval

All protocols were approved by the Office of Research Ethics at York University (e2018‐254), and in accordance with the Declaration of Helsinki. Participants provided written informed consent prior to study participation.

### 2.2 Participant description

Healthy NOC and OC women were recruited for this study. All women in the OC group were taking OC for a minimum of 3 months. All participants were 18–30 years of age, were required to have a regular menstrual cycle (cycle length: 26–30 days), and were free from a history of cardiovascular, respiratory, and hormonal conditions. Participants were instructed to refrain from fatty foods, alcoholic or caffeinated beverages, smoking, and heavy exercise for a minimum of 12 hr prior to testing. The NOC group was tested between days 2 and 5 (LH phase) and days 18 and 24 of their menstrual cycle (HH phase), whereas the OC group was tested during the placebo/no pill week (LH phase) and during their final OC pill week (HH phase). The reported OCs used included triphasic Tricyclen 28 (*n* = 2; third‐generation pill), and monophasic Cyclen 28 (*n* = 1; third‐generation pill), Yaz (*n* = 2; fourth‐generation pill), Alesse (*n* = 7; second‐generation pill), and Marvelon (*n* = 2; third‐generation pill). Menstrual cycle phase was determined using self‐report, and in the NOC group urine progesterone testing (Progesterone (PDG) urine test, Easy@Home) was conducted to confirm the presence of progesterone in the HH phase. Each participant was tested during both their LH and HH phase, except for two individuals in the OC group who only completed LH phase testing (1 each of second‐ and third‐generation pill users), creating two groups (OC and NOC) with two repeated measures (LH and HH): NOC LH (*n* = 12), NOC HH (*n* = 12), OC LH (*n* = 14), and OC HH (*n* = 12). The order of LH or HH testing sessions was randomized, but researchers were not blinded to the phase of the menstrual cycle or whether the participant was taking OC.

### 2.3 Measurements

Body mass index (BMI) was calculated as: weight (kg)/ height (m)^2^. Lower leg length (distance from tibiale to medial malleolus; mm) was estimated from height (Özaslan, İşcan, In, Tuğcu, & Koç, 2003), then lower leg volume (L) was estimated using estimated length, and measured maximum and minimum calf girth (Podleska et al., 2014). Self‐reported weekly physical activity levels and anthropometrics were used to obtain an index of VO_2_ max (mL‧kg^‐1^‧min^‐1^) using the Ainsworth equation (Ainsworth, 1992).

HR was determined using R‐R intervals from a standard single‐lead electrocardiogram (ECG; BioAMP, ADInstruments, Colorado Springs, USA). Beat‐to‐beat blood pressure was obtained using a noninvasive finger plethysmography device (BMEye Nexfin, Amsterdam, NL), and calibrated using a BPTru device (BPM 200, Coquitlam, CA). Stroke volume (SV) was also obtained with the Nexfin using pulse contour analysis (Wesseling, Jansen, Settels, & Schreuder, 1993), and Q was calculated as SV*HR/1000. Indices of SV and Q (SVi and Qi, respectively) were calculated by normalization to body surface area (BSA) according to the Dubois and Dubois formula (Du Bois & Du Bois, 1989).

Tidal volume (Vt) and respiratory rate (RR) were obtained via a heated, linear pneumotachometer (Series 3813, Hans Rudolph Inc, Shawnee Mission, USA). Ventilation (Ve) was calculated as the product of Vt and RR. A CO_2_ gas analyzer (Model 17630, Vacumed, Ventura, USA) was used to measure end‐tidal partial pressure of CO_2_ (ETCO_2_).

Heart rate variability (HRV; time domain and spectral analysis) was used as an index of parasympathetic and sympathetic activity and was determined from the ECG recording using LabChart Pro (Version 8.1.9, ADInstruments, Colorado Springs, USA). A Hann (cosine‐bell) data window was used with a window overlap of 50%, and the fast Fourier transform size was 1,024. The range for low‐frequency (LF) spectrum was 0.04–0.15 Hz and the range from the high‐frequency (HF) spectrum was 0.15–0.45 Hz. Spontaneous cardiovagal baroreceptor sensitivity (cBRS) was assessed using the sequence method (Bertinieri et al., 1988; Blaber, Yamamoto, & Hughson, 1995). For HRV and cBRS, analyses used 3–5min of data depending on trial (i.e., 3min for metaboreflex and mechanoreflex trials; 5 min for chemoreflex trial). Using the Nexfin finger pulse waveforms, and a piezoelectric pulse transducer (ADInstruments, Colorado Springs, USA) placed on the ipsilateral great toe simultaneous finger‐toe pulse wave velocity (PWV; m/s) was calculated as: (toe pulse arrival time—finger pulse arrival time)/(distance from suprasternal notch to toe—distance from suprasternal notch to finger).

### 2.4 Protocol

**Figure 1:**
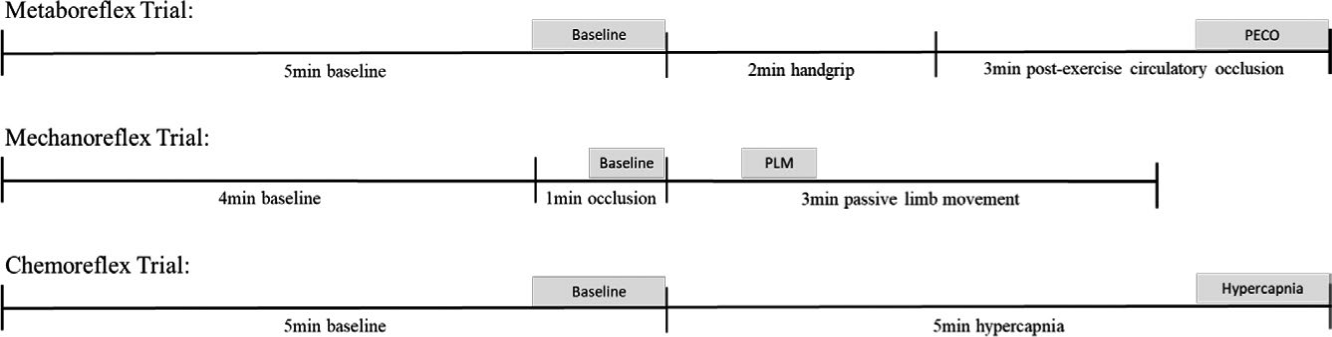
Timeline of metaboreflex, mechanoreflex, and chemoreflex trials. Shaded areas indicate where data were averaged for each time point during analysis. The order of trials was randomized

Each participant underwent three randomized assessments to separately assess the metaboreflex, mechanoreflex, and chemoreflex (Figure 1). For metaboreflex assessment, participants performed two maximum voluntary contractions (MVC) using a handgrip dynamometer (MLT004/ST Grip Force, AD Instruments, Colorado Springs, USA) at least 5min before the start of the assessment, which consisted of 5min supine rest, followed by 2 min of static handgrip exercise at 40% MVC, and 3 min postexercise circulatory occlusion (PECO). Participants were given both verbal and visual feedback to maintain 40% MVC during exercise. For PECO, a blood pressure cuff was inflated to at least 40 mmHg above systolic blood pressure (SBP) 10 s before the cessation of exercise. During mechanoreflex assessment participants laid supine for 4min then a blood pressure cuff placed distal to the right knee was inflated to at least 40mmHg above SBP for 1min to prevent fluid shifts and reduce metabolite circulation during passive leg movement (PLM) (Venturelli et al., 2016). Afterward the right leg was moved from 180 degrees extension to 90 degrees flexion at 1 Hz for 3 min. The leg blood pressure cuff was maintained at 40 mmHg above SBP for the duration of PLM, and participants were instructed both before and throughout movement to not assist or resist movement. For the chemoreflex assessment, participants laid in supine posture while breathing room air for 5 min, before breathing a hypercapnic gas mixture (5% CO_2_, 21% O_2_, and balance nitrogen) for 5 min. Each trial was separated by at least 5 min.

### 2.5 Data analysis and statistics

All signals were collected at 1000Hz using a PowerLab data acquisition system (16/35, ADInstruments, Colorado Springs, USA) and LabChart Pro software. For the metaboreflex trial, 1 min averages were obtained at the end of baseline and end of PECO. For the mechanoreflex trial, averages were taken during the last 30sec of baseline, and the second 30 s of PLM. This latter time point was used as previous work found maximal mechanoreflex changes within 1min of 1Hz PLM (Ives et al., 2013). For the chemoreflex trial, data were averaged for 1 min at the end of baseline and end of hypercapnia. Changes due to reflex stimulation were calculated as the difference between PECO/PLM/CO_2_ and baseline.

The baseline characteristics that are found in Tables 1 and 2 were averaged from the baseline data of all three trials (PECO/PLM/CO_2_). Prior to baseline averaging, one‐way repeated measures ANOVAs were completed with the a priori goal of determining any differences between the three baseline periods within 1 day of testing (i.e., phase and OC use were not factors). These comparisons are presented in the text of the Results. Once the average baseline data were obtained, these new averages were then compared between menstrual phases and OC groups using a two‐way mixed‐model ANOVA with menstrual phase as a repeated measure (Tables 1 and 2). Only baseline HRV and cBRS data are presented in Table 2 due to the perturbations of HR expected during the PECO/PLM/CO_2_ interventions. PWV analysis was conducted for the metaboreflex and chemoreflex trials only since the movement of the contralateral leg during the mechanoreflex trial moved the toe pulse transducer and adequate signals were not achieved; however, we previously noted that PWV was not affected by PLM in women (Fouladi et al., 2019). HRV, cBRS, and PWV were used as indices of autonomic, parasympathetic, and sympathetic outflow, respectively.

**Table 1:** Anthropometrics and resting cardiorespiratory and cerebrovascular dynamics

	NOC LH	NOC HH	OC LH	OC HH
Age (years)	22 ± 4		22 ± 4	
Height (cm)	163 ± 5		163 ± 7	
Weight (kg)	64 ± 10		67 ± 12	
BMI (kg‧m‐2)	24 ± 3		25 ± 4	
Lower Leg Volume (L)	2.5 ± 0.5		2.7 ± 0.7	
Predicted VO_2_ max (mL‧kg^‐1^‧min^‐1^)	37 ± 4		36 ± 2	
HR (bpm)	70 ± 5	74 ± 7†	73 ± 9	75 ± 10†
MAP (mmHg)	84 ± 5	87 ± 13	87 ± 9	89 ± 6
Qi (L‧min^‐1^‧m^‐2^)	4.1 ± 0.4	4.2 ± 0.5	4.2 ± 0.5	4.3 ± 0.6
RR (bpm)	17 ± 2	17 ± 2	17 ± 3	17 ± 3
Vt (L)	0.64 ± 0.06	0.67 ± 0.13	0.71 ± 0.19	0.73 ± 0.11
Ve (L‧min^‐1^)	11 ± 1	11 ± 2	11 ± 1	12 ± 2
ETCO_2 (_mmHg)	43 ± 2	41 ± 3†	39 ± 3*	38 ± 2†*

All values are mean ± SD.

Abbreviations: BMI, body mass index; ETCO_2,_ end‐tidal carbon dioxide; HH, high hormone; HR, heart rate; LH, low hormone; MAP, mean arterial pressure; NOC, no oral contraceptive; OC, oral contraceptive; Qi, cardiac index; RR, respiration rate; Ve, ventilation; VO_2_ max, maximum oxygen consumption; Vt, tidal volume.

^†^ Indicates a significant effect of Phase within group (*p *< .05);

* Indicates a significant effect of OC within Phase (*p *< .05).

**Table 2:** Resting autonomic function

	NOC LH	NOC HH	OC LH	OC HH
SDRR (ms)	70 ± 22	59 ± 20†	67 ± 35	64 ± 35†
RMSSD (ms)	75 ± 35	65 ± 35†	65 ± 46	61 ± 43†
pRR50 (%)	46 ± 22	40 ± 21†	36 ± 24	31 ± 24†
LF (nu)	32 ± 15	30 ± 16	34 ± 11	31 ± 9
HF (nu)	67 ± 14	69 ± 15	65 ± 11	69 ± 8
LF/HF	0.58 ± 0.40	0.54 ± 0.49	0.69 ± 0.37	0.50 ± 0.18
Total Power (µs^2^)	5246 ± 3366	3828 ± 3016†	5567 ± 8135	5519 ± 7545†
SD1 (ms)	53 ± 25	46 ± 25†	46 ± 32	43 ± 30†
SD2 (ms)	82 ± 22	69 ± 18†	81 ± 39	79 ± 41†
cBRS Slope (ms/mmHg)	31 ± 12	25 ± 7†	24 ± 11	23 ± 9†
PWV (m/s)	7.7 ± 1.3	6.7 ± 1.2†	6.6 ± 1.1	7.5 ± 1.9

All values are mean ± SD.

Abbreviations: cBRS, cardiovagal baroreceptor sensitivity; HF, high frequency; HH, high hormone phase; LF, low frequency; LH, low hormone phase; NOC, no oral contraceptive group; OC, oral contraceptive group; pRR50, proportion of RR intervals differences greater than 50 ms divided by total beats; PWV, finger‐toe pulse wave velocity; RMSSD, square root of the mean difference between adjacent beats; SD1, width of the Poincaré plot; SD2, length of the Poincaré plot; SDRR, SD between RR intervals.

^†^ Indicates a significant effect of Phase within group (*p *< .05).

**Figure 2:**
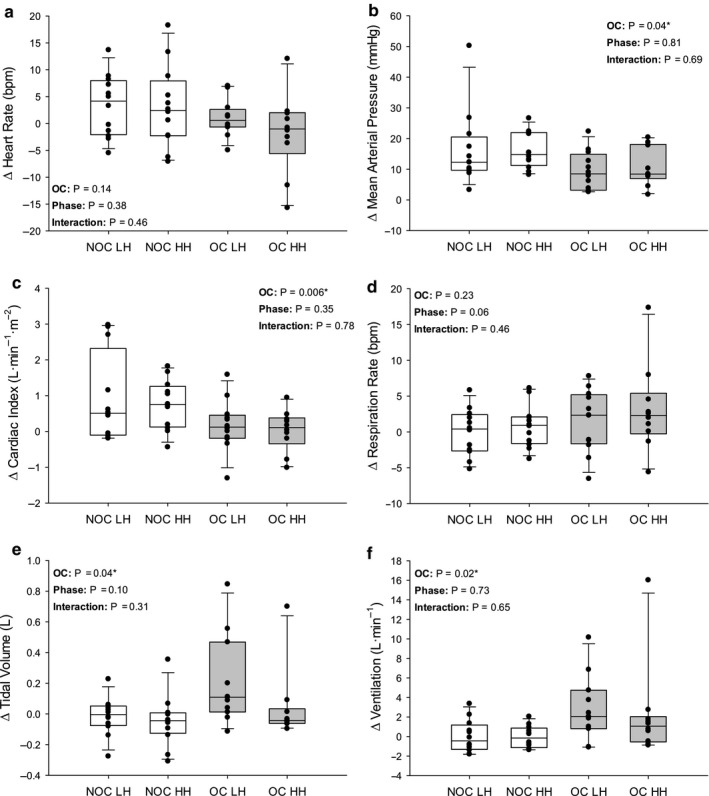
Change in cardiorespiratory dynamics in response to PECO. Change from baseline to 3rd minute of postexercise circulatory occlusion (PECO) in heart rate (a; OC LH *n* = 12; OC HH *n* = 10), mean arterial pressure (b; OC LH *n* = 12; OC HH *n* = 10), cardiac index (Figure 2c; OC LH *n* = 12; OC HH *n* = 10), respiration rate (d; OC LH *n* = 12; OC HH *n* = 10), tidal volume (Figure 2e; OC LH *n* = 11; OC HH *n* = 10), and ventilation (f; OC LH *n* = 11; OC HH *n* = 10). NOC, no oral contraceptive group; OC, oral contraceptive group; LH, low hormone phase; HH, high hormone phase. Grey bars are OC users. The line within each box indicates the median, whereas the lower and upper boundaries indicate the 25th and 75th percentile, respectively. Lower and upper whiskers indicate 10th and 90th percentile, respectively. P‐values are indicated. * indicates a significant main effect

**Figure 3:**
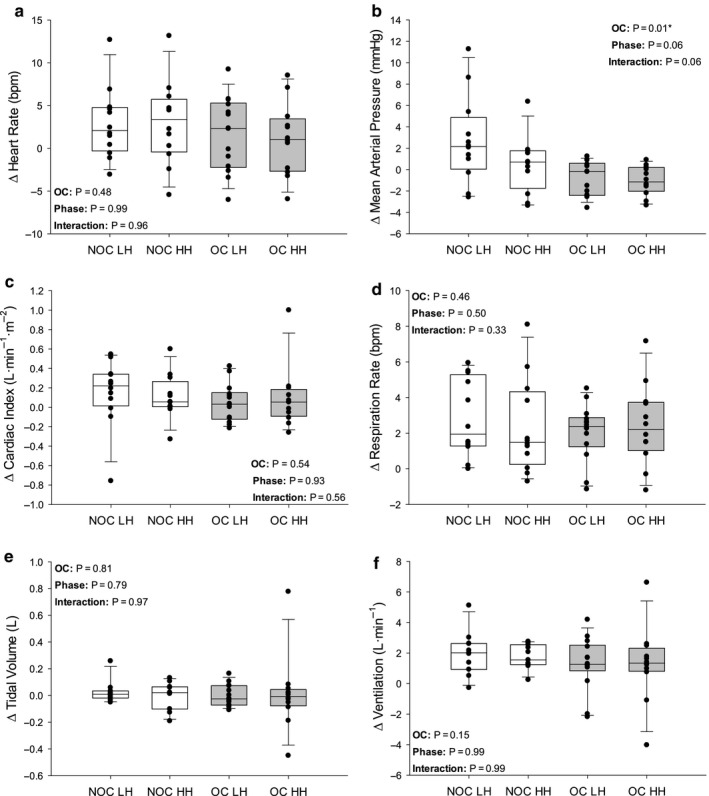
Change in cardiorespiratory dynamics in response to passive leg movement (PLM). Change from baseline to 1st minute of PLM in heart rate (a), mean arterial pressure (b), cardiac index (c), respiration rate (d), tidal volume (e; NOC LH *n* = 11; NOC HH *n* = 11), and ventilation (f; NOC LH *n* = 11; NOC HH *n* = 11). NOC, no oral contraceptive group; OC, oral contraceptive group; LH, low hormone phase; HH, high hormone phase. Grey bars are OC users. The line within each box indicates the median, whereas the lower and upper boundaries indicate the 25th and 75th percentile, respectively. Lower and upper whiskers indicate 10th and 90th percentile, respectively. *p*‐values are indicated. * indicates a significant main effect

**Figure 4:**
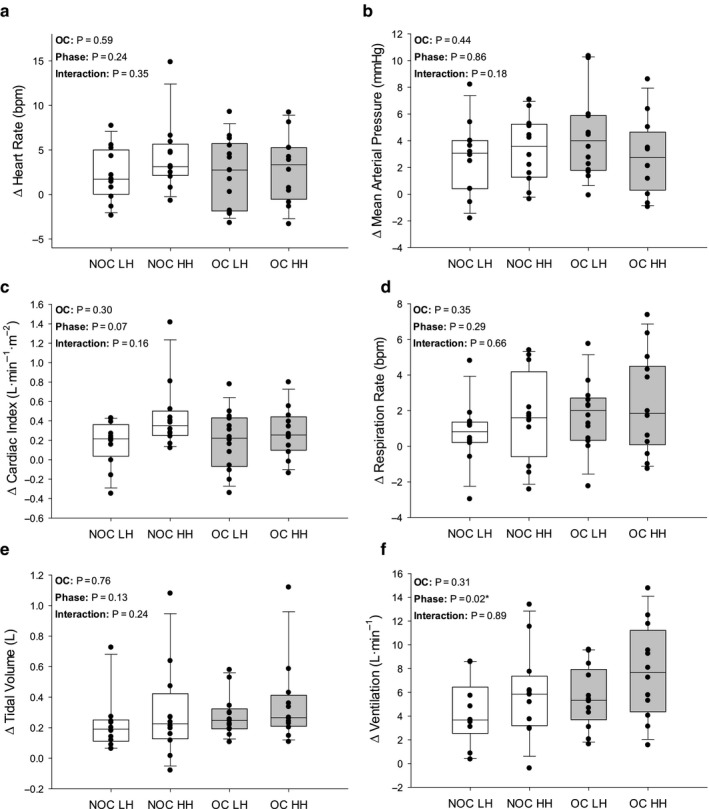
Change in cardiorespiratory dynamics in response to hypercapnia. Change from baseline to 5th minute of hypercapnia in heart rate (a), mean arterial pressure (b), cardiac index (c), respiration rate (d), tidal volume (e; NOC LH *n* = 10; OC LH *n* = 13), and ventilation (f; NOC LH *n* = 10; OC LH *n* = 13). NOC, no oral contraceptive group; OC, oral contraceptive group; LH, low hormone phase; HH, high hormone phase. Grey bars are OC users. The line within each box indicates the median, whereas the lower and upper boundaries indicate the 25th and 75th percentile, respectively. Lower and upper whiskers indicate 10th and 90th percentile, respectively. P‐values are indicated. * indicates a significant main effect

Changes in cardiorespiratory dynamics during each trial (Figures 2, 3, 4) were investigated using 2‐way mixed‐model ANOVAs including menstrual phase as a repeated‐measures factor and OC use as a non–repeated measures factor. Tukey's Honestly Significant Difference was used to *post hoc* test significant interactions. To test the changes in PWV due to PECO or PLM (text of Results), three‐way mixed‐model ANOVAs were conducted to determine any main and interaction effects among OC use, menstrual phase, and trial. Phase and trial were repeated measures. Least square means for significant main effects were determined and presented in the text. Statistical significance was set a priori at *p* ≤ .05 for all tests. All one‐ and two‐way mixed‐model comparisons were completed using Sigmaplot 13.0 (Systat Software Inc, San Jose, USA), and the three‐way mixed‐model comparison was conducted using IBM SPSS Statistics 23 (Armonk, New York, USA).

In text and tables, data are displayed as mean ± standard deviation, whereas data in figures are presented as median with interquartile range and 10th and 90th percentile. *n* values are indicated for each group only if a full complement of participants was not available for an outcome.

## 3 RESULTS

### 3.1 Baseline comparisons

When comparing the baseline data between the three trials (prior to averaging for Tables 1 and 2), the chemoreflex baseline differed from the mechanoreflex and metaboreflex trials. For the NOC‐LH group, Qi, Vt, and ETCO_2_ were higher in the chemoreflex baseline compared to the mechanoreflex and metaboreflex trials (*p* < .03), and HR and Ve were higher in the chemoreflex baseline compared to only the mechanoreflex baseline (*p* < .03). For the NOC‐HH group, ETCO_2_ and Vt were higher, and RR was lower, in the chemoreflex baseline compared to the mechanoreflex and metaboreflex trials (*p* < .05). For the OC‐LH group, Vt and Ve were higher, and RR was lower, in the chemoreflex baseline compared to the other trials (*p* ≤ .05). For the OC‐HH group, HR, ETCO_2_, Vt, and Ve were higher in the chemoreflex baseline compared to the other trials (*p* < .02). There were no differences between trials in any group for HRV, cBRS, or PWV (*p* > .05).

During the HH phase, the OC group achieved a lower percentage of MVC compared to their LH phase (33 ± 5% vs. 36 ± 3%, *p* = .040) and the HH phase of the NOC group (33 ± 5% vs. 37 ± 3%, *p* = .008), but not the LH phase of the NOC group (36 ± 3% vs. 36 ± 3%, *p* = .686). There was no difference between OC and NOC groups for age (*p* = .827), height (*p* = .898), weight (*p* = .412), BMI (*p* = .354), lower leg volume (*p* = .219), or predicted VO_2_ max (*p* = .483). Resting HR was higher in the HH phase, compared to the LH phase (*p* = .032), but there was no effect of OC use (*p* = .553). There was no effect of phase (*p* = .247) or OC use (*p* = .336) on MAP, and neither phase (*p* = .536) nor OC use (*p* = .565) affected Qi. Similarly, there were no phase or OC use effects for RR (*p* = .790 and *p* = .411, respectively), Vt (*p* = .116 and *p* = .294, respectively), or Ve (*p* = .085 and *p* = .142, respectively). ETCO_2_ was lower in the OC group compared to the NOC group (*p* < .001), and lower in the HH phase compared to the LH phase (*p* = .033; Table 1).

The standard deviation among RR intervals (SDRR), square root of the mean difference between adjacent beats (RMSSD), and proportion of RR interval differences greater than 50ms divided by total beats (pRR50) were lower in the HH phase compared to the LH phase (*p* = .007, *p* = .016, and *p* = .007, respectively), with no effect of OC use (*p* = .997, *p* = .558, and *p* = .218, respectively). There were no differences in low‐frequency (LF) power, high‐frequency (HF) power, or LF/HF due to menstrual phase (*p* = .403, *p* = .318, and *p* = .307, respectively) or OC use (*p* = .583, *p* = .684, and *p* = .549, respectively). Total power was lower in the HH phase compared to the LH phase (*p* = .016) with no effect of OC use (*p* = .762). Similarly, both the width (SD1) and length (SD2) of the Poincaré plot were lower in the HH phase (*p* = .015 and *p* = .008, respectively), but were not different due to OC use (*p* = .561 and *p* = .753, respectively). cBRS slope was lower in the HH phase (*p* = .012) with no effect of OC use (*p* = .264). PWV decreased due to menstrual phase in the NOC group, but not with OC use (*p* = .012; Table 2).

### 3.2 Metaboreflex trials

During PECO, there were no phase (*p* = .376) or OC (*p* = .137) effects for Δ HR (Figure 2a). Δ MAP was not different between the LH and HH phases (*p* = .810) but was lower in OC users compared to the NOC group (*p* = .043; Figure 2b). Similarly, while there was no phase effect (*p* = .345), Δ Qi was lower in OC users compared to the NOC group (*p* = .006; Figure 2c). There was no significant effect of group (*p* = .228) or phase (*p* = .061) for Δ RR. In the OC group Δ Vt was greater, compared to the NOC group (*p* = .042), but there was no difference between phases (*p* = .096; Figure 2e). Importantly, Δ Ve was greater in the OC users compared to the NOC group (*p* = .024) but was not different between phases (*p* = .732; Figure 2f). PWV was significantly increased by HG and PECO compared to baseline (*p* < .001; Baseline: 7.02 ± 1.42m/s; HG: 7.84 ± 1.42cm/s; PECO: 8.14 ± 1.47m/s) with no effect of OC or Phase.

### 3.3 Mechanoreflex trials

In response to PLM, there were no phase (*p* = .994) or OC use (*p* = .477) effects on Δ HR (Figure 3a). Similarly, there was no significant effect of phase on Δ MAP (*p* = .057), however, Δ MAP was lower in the OC users compared to the NOC group (*p* = .012; Figure 3b). Δ Qi was not affected by phase (*p* = .929) or OC use (*p* = .536) nor were there effects of phase or OC use on Δ RR (*p* = .500 and *p* = .459, respectively; Figure 3d), Δ Vt (*p* = .786 and *p* = .814, respectively; Figure 3e), or Δ Ve (*p* = .993 and *p* = .151, respectively; Figure 3f).

### 3.4 Chemoreflex trials

During CO_2_ chemoreflex assessment, there were no phase or OC use effects for Δ HR (*p* = .241 and *p* = .589, respectively; Figure 4a), Δ MAP (*p* = .858 and *p* = .436, respectively; Figure 4b), or Δ Qi (*p* = .069 and *p* = .300, respectively; Figure 4c). There were no effects of phase or OC use for Δ RR (*p* = .286 and *p* = .354, respectively; Figure 4d) or Δ Vt (*p* = .128 and *p* = .755, respectively; Figure 4e); however, Δ Ve was greater in the HH, compared to the LH phase (*p* = .022) with no effect of OC use (*p* = .310). CO_2_ exposure significantly increased PWV (Baseline: 7.13 ± 0.75 to CO_2_: 7.45 ± 0.75; *p* = .023), and there was a significant interaction between OC and menstrual phase (*p* = .002) where PWV was lower in the OC LH phase compared to the NOC LH phase (*p* = .015) (NOC‐LH: 7.97 ± 1.46 m/s; NOC‐HH: 6.92 ± 1.46 m/s; OC‐LH: 6.71 ± 1.35 m/s; OC‐HH: 7.58 ± 1.46 m/s).

### 3.5 End‐tidal CO_2_ measurements

All groups exhibited similar reductions in ETCO_2_ in response to both PECO and PLM (*p* > .05; data not shown). The Δ ETCO_2_ in response to hypercapnia was greater in the OC users’ LH phase compared to that of the NOC group (*p* = .029; NOC‐LH: +6.6 ± 1.8mmHg; NOC‐HH: +7.3 ± 1.7mmHg; OC‐LH: +8.1 ± 1.9mmHg; OC‐HH: +7.3 ± 1.5mmHg).

## 4 DISCUSSION

This study aimed to describe hormonal effects on the hemodynamic and ventilatory responses to the activation of the mechanoreflex, metaboreflex, and CO_2_ chemoreflex in women. Our most interesting findings were that (a) women taking oral contraceptives displayed an attenuated pressor response to both metaboreflex and mechanoreflex activation; however, they had an augmented ventilatory response to metaboreflex activation, (b) women in the high hormone phase of the menstrual cycle exhibited greater ventilation during hypercapnia exposure, and (c) at rest women in the high hormone phase of the menstrual cycle exhibited higher HR, lower HRV, and reduced parasympathetic control of heart rate.

### 4.1 Metaboreflex and mechanoreflex

Sex differences in both the metaboreflex and the mechanoreflex have been previously noted (Ives et al., 2013; Jarvis et al., 2011) and differences are often attributed to female sex hormones. However, while studies of metaboreflex function observed no hemodynamic or sympathetic differences throughout the menstrual cycle (Hartwich, Aldred, & Fisher, 2013; Jarvis et al., 2011), these investigations did not investigate OC use or ventilatory responses. Furthermore, no mechanoreflex studies had previously considered the role of the menstrual cycle or OC use on any cardiorespiratory variable. This is surprising considering the documented hyperventilatory effects of progesterone (Skatrud, 1989) and the synergistic effect of progesterone and estrogen in increasing ventilation (Shahar et al., 2003).

Our findings highlight that OC use can influence the cardiorespiratory responses to reflex activation. OC use improves endothelial‐dependant vasodilation (Meendering, Torgrimson, Miller, Kaplan, & Minson, 2010; Simoncini et al., 2007), which might be attributable to an estrogen‐induced increase in nitric oxide bioavailability (Cicinelli et al., 1999; Kleinert et al., 1998), but perhaps more likely due to be greater ß2‐adrenergic receptor‐mediated vasodilation (Limberg et al., 2016). Dilation of pulmonary bronchioles is also mediated by ß2‐adrenergic receptors (Barnes, 1986), and previous work has documented an augmented relaxation of bronchiole smooth muscle in response to a ß agonist with concomitant progesterone administration (Foster, Goldie, & Paterson, 1983). We suggest that greater β2‐receptor–mediated vascular and bronchiole dilation in OC users during the adrenergic stimulus of PECO is contributing to the observed attenuated blood pressure response and the enhanced ventilatory response. We further suggest that the greater ventilatory response to reflex stimulation in OC users was only evident during PECO due to a sufficient sympathetic and adrenergic response to influence the pulmonary system. While not conclusive, yet in partial support of this, finger‐toe PWV increased more during PECO compared to 5% CO_2_ (with no previously demonstrated increase in PWV during PLM (Fouladi et al., 2019)), perhaps suggesting greater sympathetic outflow during PECO. However, MAP was not concurrently enhanced during PECO in OC users, in fact it was attenuated. As it has been observed that young women may display an attenuated relationship between peripheral resistance and sympathetic activity (Baker, Limberg, Ranadive, & Joyner, 2016; Joyner, Barnes, Hart, Wallin, & Charkoudian, 2015), we hypothesize that while there is more sympathetic activity during metaboreflex activation, which is eliciting some peripheral vasoconstriction (thus, the increase in PWV), we did not observe an increase in MAP due to reduced neurovascular transduction, and potentially more vasodilation in other vascular beds. We recommend measuring catecholamines, muscle sympathetic nerve activity, and regional blood flow in future studies.

### 4.2 Chemoreflex

There was no influence of OC use on chemoreflex function, yet there was a significant effect of the menstrual cycle on the ventilatory response to hypercapnia where women in the HH phase had an exacerbated response. This was true if the cycling sex hormones were endogenous or pharmaceutical. Interestingly, Usselman et al. previously found that in women taking OC, the sympathetic response to chemoreflex activation via apnea was greater in the LH phase (Usselman et al., 2013). Since apnea also includes activation of both pulmonary stretch receptors and hypoxia, we suggest that the sympathetic activation in the LH phase observed by Usselman et al. was caused by one of these alternate reflex pathways rather than hypercapnia. However, Richalet et al. recently found that there was no influence of OC use on the ventilatory or heart rate response to whole‐body hypoxic exercise (Richalet, Lhuissier, & Jean, 2020). Little information could be found on the effect of OC use on pulmonary stretch receptors.

Contrary to our current findings, Hazlett and Edgell found no significant menstrual cycle phase differences in the ventilatory response to hypercapnia (Hazlett & Edgell, 2018). However, the analysis of those data did not isolate the response to hypercapnia from the response to upright tilt, and did not include OC users and, thus, the current study had a greater sample size of women. Similar to the current study, Macnutt et al. found that women in the mid‐luteal phase (i.e., HH phase) had a lower ventilatory recruitment threshold during hyperoxic hypercapania implying an earlier ventilatory response (Macnutt, De Souza, Tomczak, Homer, & Sheel, 2012). Together, our findings suggest that the presence of endogenous or pharmaceutical female sex hormones can influence the ventilatory response to hypercapnia. Supportive of this, multiple researchers have found a greater ventilatory stimulus during maximal exercise tests in women during the HH phase. Schoene et al. found that the ventilatory drive during a maximal cycling test was greater in women during the HH phase (Schoene, Robertson, Pierson, & Peterson, 1981). Vaiksaar et al. similarly found a greater ventilatory equivalent (Ve/VCO_2_) in the HH phase of female rowers taking OC (Vaiksaar et al., 2011). Lastly, Smekal et al. also noted a greater Ve/VCO_2_ during a cycle ergometer test to voluntary exhaustion in eumenorrheic women in the HH phase (Smekal et al., 2007).

### 4.3 Influence of OC and menstrual cycle at rest

In the current study, we noted that women taking OC had lower resting ET‐CO_2_ compared to those who were not taking OC. Our group has also previously noted lower ET‐CO_2_ in OC users (Abidi et al., 2017). Interestingly, we did not observe an effect of OC on resting ventilatory measurements. As hypothesized earlier, we suggest that OC use upregulates β2receptors on both the vasculature and bronchioles which could increase gas exchange at the level of the pulmonary capillary thus decreasing ET‐CO_2_. Furthermore, OC users have been shown to have greater hemoglobin (Mattu et al., 2020) which could also be contributing to their enhanced gas exchange. During supine rest, we also noted that women in the HH phase of the menstrual cycle had higher HR, lower HRV, and lower ET‐CO_2_. Our group and others have also found that HR is greater in the HH phase of the menstrual cycle (Abidi et al., 2017; Usselman et al., 2016), and that resting sympathetic activity is higher in the HH phase of the menstrual cycle (Fu et al., 2009; Usselman et al., 2016) supporting our current findings. However, we also noted that the PWV of NOC users was lower in the HH phase perhaps indicating less peripheral vasoconstriction in the presence of endogenous (but not pharmaceutical) female sex hormones. While finger‐toe PWV has been shown to correlate with carotid‐radial PWV (Edgell, Stickland, & MacLean, 2016), it has not been correlated with more traditional measures such as carotid‐femoral PWV or with direct measures of sympathetic activity. Lastly, ET‐CO_2_ was observed to be lower in the HH phase of the menstrual cycle. We did not observe a concurrent increase in ventilation, yet many other groups have made this observation (Hazlett & Edgell, 2018; Macnutt et al., 2012; Schoene et al., 1981) and we may have experienced type II error. An increase in ventilation could be directly responsible for the reduction in ET‐CO_2_.

### 4.4 Limitations

Importantly, combining multiple generations of OC pill use could have confounded our results. Indeed, Shenouda et al. recently found that in second‐generation pill users (but not in other pill generation users), there was a negative association between OC use duration and flow‐mediated dilation (Shenouda, Priest, Rizzuto, & MacDonald, 2018). Unfortunately, we do not have the necessary sample size to compare the pill generations in the current study, and therefore, we recommend larger sample sizes for future studies. Furthermore, quantitative measurements of estrogen and progesterone analogs could have been helpful for this study. However, Shenouda et al. also found that serum immunoassays did not detect estrogen or progesterone in samples from OC users above that observed in men or women in the early follicular phase of the menstrual cycle. Thus, we further recommend that future studies involving OC users should include high‐performance liquid chromatography analysis of all analogs of estrogen and progesterone (both endogenous and pharmaceutical), as developed recently (Blue et al., 2018).

We found that there were differences between the baseline period of the chemoreflex trial compared to the baseline periods of both the mechanoreflex and metaboreflex trials; however, there was no difference between the latter two trials. Primarily these findings showed hyperventilation (possibly due to higher ETCO_2_) and increased HR. Since trials were randomized, we suggest that these differences are due to the anticipation of breathing hypercapnic gas, an unknown circumstance for most people. Indeed, Tobin et al. found that in periods of exercise anticipation or mental stress, participants underwent both hyperventilation and greater CO_2_ production (Tobin, Perez, Guenther, D'Alonzo, & Dantzker, 1986). While we could not blind the participants to the trial that they were undergoing, we suggest that future investigations run multiple gas trials to potentially eliminate this phenomenon. However, despite this slightly stimulated state, we still observed the expected increases in cardiorespiratory variables in response to hypercapnia in all groups.

While BMI was measured and leg volume was estimated, muscle mass measurements were not conducted and muscle fiber type differences were not determined, both of which would influence metabolite build‐up and therefore the metaboreflex response. Of note, the OC users in the HH phase could not maintain the same level of handgrip force despite encouragement and visual feedback, perhaps indicating more fatiguability. Previously, our group observed that OC users had greater postural sway at the end of a 10‐min standing test (Abidi et al., 2017). These observations suggest that studies investigating muscular fatigue in OC users are warranted.

Not only does hypercapnia exposure influence the central chemoreceptors, but it is also detected by the peripheral chemoreceptors. We did not conduct hypoxia trials to assess peripheral chemosensitivity nor did we measure ETO_2_ levels. However, we previously observed an increase in ETO_2_ of approximately 20mmHg in men and women in the LH and HH phases of the menstrual cycle during hypercapnia (without sex or menstrual phase differences)(Hazlett & Edgell, 2018) suggesting arterial hyperoxia which would attenuate peripheral chemoreflex activation. Lastly, this was a cross‐sectional study comparing women who were not taking oral contraceptives to those who were. While this is an important first step in determining the role of oral contraceptives on autonomic reflex function, longitudinal studies which follow women from before prescription to afterwards are needed.

## 5 C**ONCLUSIONS**

The pressor response to activation of the mechanoreflex and metaboreflex is attenuated in OC users; however, the ventilatory response to metaboreflex activation was enhanced in OC users. We suggest that chronic upregulation of β‐adrenergic receptors may play an important role. While there was no influence of the menstrual cycle on the responses to either metaboreflex or mechanoreflex activation, women in the HH phase had a significantly greater ventilatory response to hypercapnia suggesting that both endogenous and pharmaceutical estrogen and progesterone can influence the CO_2_ chemoreflex in women. Lastly, at rest women in the HH phase of the menstrual cycle had higher HR, and reduced HRV indices (potentially due to lower parasympathetic control of HR) compared to the LH phase suggesting a change in resting autonomic balance.

## CONFLICT OF INTERESTS

There are no conflicts of interest to disclose.

## AUTHOR CONTRIBUTIONS

All experiments were performed in the laboratory of HE. EA, II, SW, and HE contributed to the conception and design of the work. EA, II, SW, BA, KM, and HE contributed to the acquisition, analysis, or interpretation of data for the work. EA, II, SW, BA, KM, and HE contributed to drafting the work or revising it critically for important intellectual content. All authors approved the final version of the manuscript, agree to be accountable for all aspects of the work, and ensure that questions related to the accuracy or integrity of any part of the work are appropriately investigated and resolved. All persons designated as authors qualify for authorship, and all those who qualify for authorship are listed.
